# Focusing on Digital Research Priorities for Advancing the Access and Quality of Mental Health

**DOI:** 10.2196/47898

**Published:** 2023-04-24

**Authors:** John Torous, Nicole M Benson, Keris Myrick, Gunther Eysenbach

**Affiliations:** 1 Beth Israel Deaoness Medical Center Boston, MA United States; 2 McLean Hospital Belmont, MA United States; 3 Inseperable Washington, DC United States; 4 JMIR Publications Toronto, ON Canada

**Keywords:** digital phenotyping, mental health, depression, anxiety, smartphone

## Abstract

Digital mental health solutions are now well recognized as critical to solving the global mental health crisis. As research accelerates, it is now clear that solutions ranging from computer-based therapy programs to virtual reality headsets and smartphone apps to large language model chatbots are of interest, feasible, and hold exciting potential to improve mental health. This research should now consider the next generation of scientific and clinical questions regarding if these new approaches are equitable, valid, effective, implementable, efficacious, and even cost-effective. This paper outlines several of the new frontiers for the next generation of research and introduces JMIR Publications’ partnership with the Society of Digital Psychiatry to further advance these aims.

With the rapid acceleration of digital mental health, there is a growing need to ensure that the field delivers on its potential to increase access and quality of care. While the initial phase of research has already yielded important insights, it has also highlighted key challenges and areas that require further attention. Rather than providing a comprehensive overview of the field, the aim of this paper is to emphasize specific aspects of digital mental health that may be of particular interest as we move forward.

Equity in digital health has emerged as a key concern, as early assumptions that digital mental health tools would easily reach and serve all people have been replaced with the realization that several digital divides exist, especially around digital access, literacy, and skills. Therefore, addressing digital literacy and skills barriers may represent the single most important priority for ensuring that the full potential of digital health can be harnessed to benefit people with the greatest needs. It will also be important to explore the role of digital technology as a new social determinant of health as well as a tool to support addressing social determinants of mental health.

While studies that showcase the effectiveness of digital tools or interventions in single use cases are important, those demonstrating their multisite and global applications are particularly relevant. Adapting and tailoring digital tools to suit the needs of diverse cultural and regional contexts is still relatively uncommon, despite the potential for significant impact. Research focused on programs that can be scaled to serve diverse communities and provide care to underserved populations is therefore of great interest. Likewise, examining how ideas can flow in both directions and new solutions can emerge from all regions can yield valuable insights and promote more equitable access to digital health worldwide. Relatedly, equity in the voices of researchers and authors remains a priority, and *JMIR Mental Health* will continue its “Patient Perspective” pieces to offer a direct voice to those with lived experience.

Ensuring replicability is a cornerstone of all scientific research, and digital mental health research is no exception. Studies examining the impact of proprietary tools that are not accessible to others pose a significant threat to reproducible science. While such research is still valuable, authors must explore creative solutions that allow others to engage with their approach and build on their findings. Similarly, to promote transparency and minimize potential bias, statistical and machine learning models should be presented in a clear and accessible manner that enables replication and assessment of bias. Relatedly, addressing challenges around missing data, especially from digital phenotyping research, and assumptions in preprocessing data are now critical for the success of this work. Preregistration of models, use of open-source tools, and data sharing are essential measures that will become increasingly critical to the field. Validation with biological markers will become more feasible and relevant, but validation through practical use and real-world positive outcomes will always be the most relevant.

Privacy is a top concern for the digital health space, particularly in the realm of digital mental health. While research that highlights this concern is valuable in measuring progress, papers that present novel solutions to these privacy issues are of even greater interest. Topics that are likely to have a high impact include efforts to increase public awareness and engagement around privacy as well as new regulatory proposals to enhance privacy protections. Relatedly, ethics in research remains equally salient, and questions about the informed nature of consent based solely on legalese buried in privacy policies will continue to raise concerns.

Efficacy is a key focus of the digital mental health space, and there is a wide range of claims about the clinical impact of various interventions. However, it has become clear that the level of human support offered can significantly impact the efficacy of these interventions. Therefore, research that assesses the optimal degree and mode of human support necessary to make interventions more effective is of great interest. Additionally, claims of efficacy should likely be compared against an active digital control intervention to ensure their rigor and relevance. While research without adequate control groups can help refine interventions and provide important feasibility data, it is less likely to contribute to a deeper understanding of efficacy. Likewise, retrospective studies of select cohorts of certain patient populations are interesting, but prospective studies are often the most informative.

As concerns about low engagement with digital health tools have become more apparent, there is an urgent need for innovative solutions. Studies that fail to provide clear insights into engagement present a significant challenge to the field, and transparent reporting has become more critical than ever. As noted earlier, providing human support can play a crucial role in enhancing engagement, and it is also important to focus on research that investigates design factors and adaptive interventions with context awareness. Furthermore, with growing recognition that clinician or coach support is vital for patient engagement, research on the adoption of clinical systems and reports on their implementation in care settings have become especially relevant and timely. Beyond engagement, research that explores the extent to which digital technologies impact client activation—clients taking an active role in treatment and maximizing shared decision-making between provider and client to move toward recovery outcomes—is also a needed area of focus.

As the field of digital health continues to grow, so too will the areas of research prioritization. Given the vast and dynamic nature of this field, there will always be exceptional topics that need to be covered despite any abovementioned concerns. For example, cross-sectional studies of interest in digital mental health have already been well reported on, but with new advances in large language models and related artificial intelligence language models, it will be important to understand interest, concerns, and potential even before more advanced research. New voices need to be heard and innovative pilot studies run with new advances in these language models. However, our collective goal remains the same: to highlight only the highest quality research. To achieve this goal, papers should continue to emphasize the clinical relevance of their findings and explain how their work can help advance access and quality of care.

Finally, as the field expands, there is a need to bring new voices into the field and transform research findings into practice. To accomplish these goals, JMIR Publications has partnered with the Society of Digital Psychiatry to make *JMIR Mental Health* the official society journal. As the society advances standards and advocates for high-quality practices/standards, the journal will serve to convene stakeholders and disseminate consensus papers. By bridging academics, industry, patient, clinician, regulatory, and family voices, the synergy of *JMIR Mental Health* and the Society of Digital Psychiatry will together help advance the mission of increased access and quality of mental health services for all.

